# Epigenetic control of influenza virus: role of H3K79 methylation in interferon-induced antiviral response

**DOI:** 10.1038/s41598-018-19370-6

**Published:** 2018-01-19

**Authors:** Laura Marcos-Villar, Juan Díaz-Colunga, Juan Sandoval, Noelia Zamarreño, Sara Landeras-Bueno, Manel Esteller, Ana Falcón, Amelia Nieto

**Affiliations:** 1 Centro Nacional de Biotecnología, (CNB-CSIC), Darwin 3, Cantoblanco, 28049 Madrid, Spain; 2 Ciber de Enfermedades Respiratorias, Madrid, Spain; 3 Cancer Epigenetics and Biology Program (PEBC), Bellvitge Biomedical Research Institute (IDIBELL), Gran Vía 199, Hospitalet de Llobregat, 08908 Barcelona, Catalonia Spain; 4 Biomarkers and Precision Medicne Unit (UByMP), Instituto de Investigación Sanitaria La Fe (IISLaFe), Avda Fernando Abril Martorell, 106, 46026 Valencia, Spain; 5 Physiological Sciences Department, School of Medicine and Health Sciences, University of Barcelona (UB), Catalonia, Spain; 6 Institucio Catalana de Recerca i Estudis Avançats (ICREA), Barcelona, Catalonia Spain

## Abstract

Influenza virus stablishes a network of virus-host functional interactions, which depends on chromatin dynamic and therefore on epigenetic modifications. Using an unbiased search, we analyzed the epigenetic changes at DNA methylation and post-translational histone modification levels induced by the infection. DNA methylation was unaltered, while we found a general decrease on histone acetylation, which correlates with transcriptional inactivation and may cooperate with the impairment of cellular transcription that causes influenza virus infection. A particular increase in H3K79 methylation was observed and the use of an inhibitor of the specific H3K79 methylase, Dot1L enzyme, or its silencing, increased influenza virus replication. The antiviral response was reduced in conditions of Dot1L downregulation, since decreased nuclear translocation of NF-kB complex, and IFN-β, Mx1 and ISG56 expression was detected. The data suggested a control of antiviral signaling by methylation of H3K79 and consequently, influenza virus replication was unaffected in IFN pathway-compromised, Dot1L-inhibited cells. H3K79 methylation also controlled replication of another potent interferon-inducing virus such as vesicular stomatitis virus, but did not modify amplification of respiratory syncytial virus that poorly induces interferon signaling. Epigenetic methylation of H3K79 might have an important role in controlling interferon-induced signaling against viral pathogens.

## Introduction

Chromatin is a dynamic structure that adapts itself to alter the spatial arrangement of genetic information and thus meet the changing demands of cell functions; it has a key role in the control of gene expression. Epigenetic modifications of chromatin regulate heritable and reversible gene expression without altering the DNA sequence, and can also modify chromatin accessibility. DNA methylation and post-translational histone acetylation and methylation are major mechanisms responsible for epigenetic regulation of gene expression^1^. DNA methylation typically represses gene transcription when takes place in the promoters of regulated genes^2^. Histone acetylation opens the condensed chromatin structure by reducing DNA affinity for histones; this enables the DNA to uncoil from nucleosomes so that transcription factors and RNA polymerase can access the DNA and increase transcription^3^. Histone methylation can increase or decrease gene transcription, depending on which amino acids in the histones are modified and how many methyl groups are added to specific residues^4^.

Influenza virus is an RNA virus whose genome consists of eight single-stranded RNA segments with negative polarity. The virus uses a very uncommon transcription mechanism. The heterotrimeric viral polymerase synthesizes capped, polyadenylated viral mRNAs through an initiation process; it uses short-capped oligonucleotides as primers, which are scavenged by a viral endonuclease from newly synthesized host RNA polymerase II transcripts^5,6^. The viral transcription strategy thus requires continuous cellular transcription for viral mRNA synthesis. This mechanism implies functional association with host genome expression, and thus depends on chromatin dynamics. Despite this requisite, once viral transcription is complete, cellular transcription is no longer needed and the virus efficiently switches off host mRNA synthesis through a complex interplay of processes^7^. The infected cell in turn initiates an antiviral response to counterattack the infection by inducing different pathways, among them the interferon (IFN)-stimulated gene (ISG) response. Hundreds of ISGs with antiviral and immune modulatory functions are transcribed after influenza virus infection^8^. During infection, host cell chromatin dynamics must respond to various situations, to increase or reduce expression of specific genes that adapt the infected cell for the host:pathogen confrontation at each replication.

There is little information about epigenetic changes elicited by influenza virus infection in host cells. Changes in total DNA methylation are observed during infection with the very pathogenic H5N1 virus in the thymus of infected chickens^9^ or in some inflammatory genes in human lung epithelial A549 infected cells^10,11^. Downregulation of histone deacetylase 6 activity in MDCK cells is reported^12^, as are changes in the histone methylation status of several ISGs in human respiratory cells infected with an H1N1 strain^8^.

Here we performed an unbiased screening of epigenetic changes in influenza virus-infected cells, both at DNA and histone modification levels. Whereas DNA methylation was unaltered, we observed specific changes in the methylation status of lysine 79 of histone 3 (H3K79). Use of a specific inhibitor of H3K79 methylase or its silencing showed that methylation of this residue has a prominent role in the control of metabolic pathways involved in counteracting infection by several viral pathogens via interferon signaling.

## Results

### Epigenetic changes induced by influenza virus replication

#### DNA methylation in influenza virus infected cells

**Figure 1:**
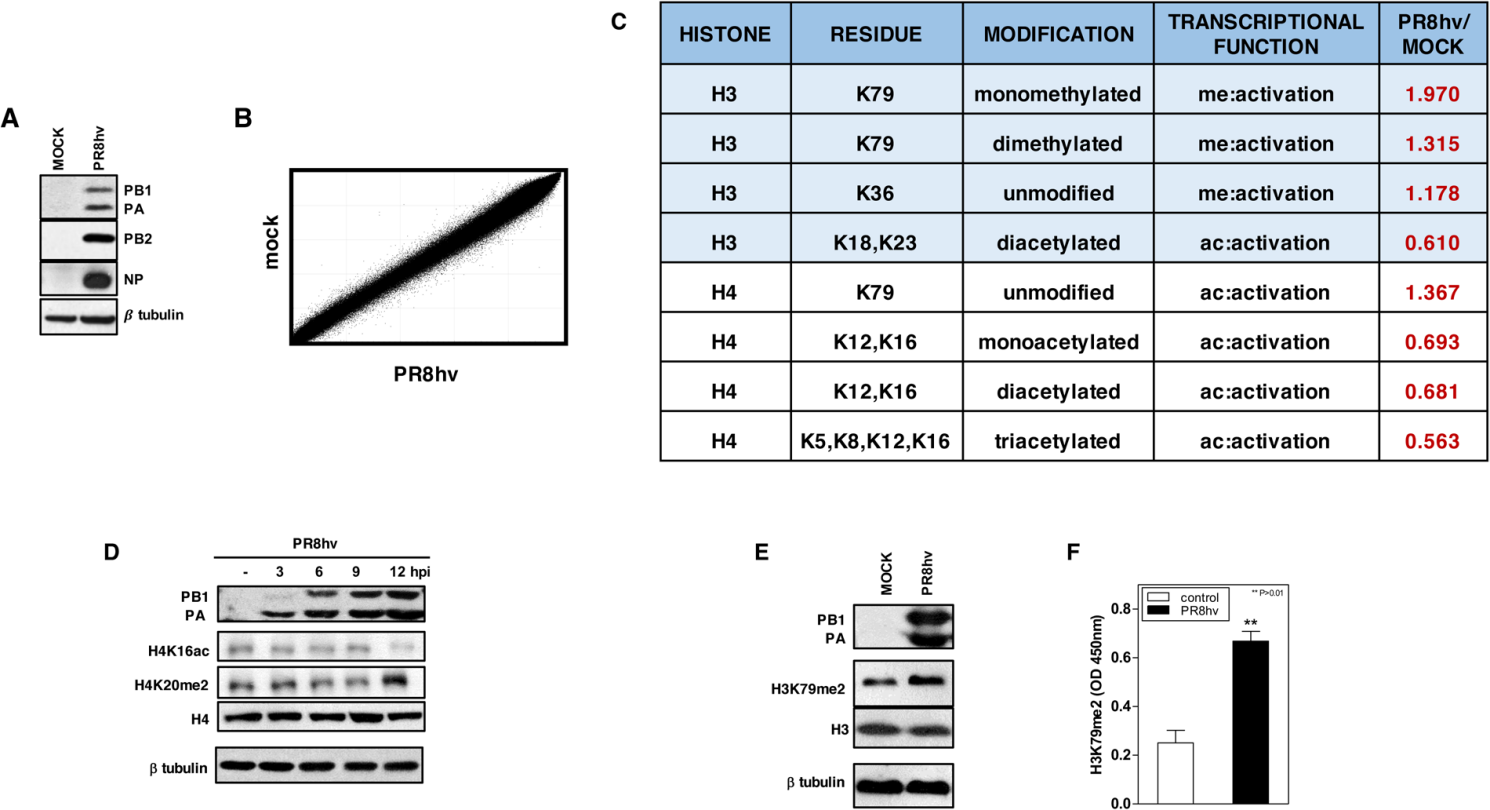
Influenza virus infection alters post-translational modifications of histones. A549 cells were mock-infected or PR8hv-infected (m.o.i. 3) and various fractions were obtained at 8 hpi. (**A**) Western blot analysis showing PB1, PA, PB2 and NP viral proteins and β-tubulin in total cell extracts. (**B**) DNA was isolated and used to detect DNA methylation. Representative scatter plot showing CpG methylation levels in mock- and influenza virus-infected cells. (**C**) In parallel, the histone fraction from mock- or influenza virus-infected cells was purified and analyzed for PTM using the nanoLC-MS platform. The modification column indicates the assigned function of each PTM; ac, acetylation; me, methylation. The red column (right) shows PR8hv fold change relative to mock-infected cells for each PTM. (**D**) Western blot analysis against the indicated proteins collected at different times post-infection. (**E**,**F**) Histones from mock- or PR8hv-infected cells were purified and used for Western blot analysis to detect the indicated proteins (**E**) or for colorimetric determination (**F**) of dimethylated H3K79. DNA methylation was analyzed in duplicate; proteomic analysis was performed in triplicate.

To identify epigenetic changes induced by influenza virus infection in the cell transcription machinery, we first analyzed DNA methylation levels. Human A549 lung epithelial cells were mock-infected or infected at high multiplicity of infection (m.o.i.; 3 PFU/ml) with a hypervirulent A/PR8/8/34 (PR8hv) strain^13–16^ (Fig. 1A). At 8 h post infection (hpi), DNA was isolated and methylation profiles evaluated in triplicate using the 450 K Infinium DNA Methylation BeadChip. Correlation analysis of the 444,751 valid probes showed an extremely strong relationship between samples (Pearson correlation coefficient from R2 = 0.9945 to R2 = 0.9960; Fig. 1B). To identify specific differentially methylated candidates, we performed a parametric analysis to compare average beta values from infected with mock-infected cells, selecting those with differences in methylation levels >25 (−0.25>delta>0.25) and a standard deviation value <10% (Desvest<0.10). We found no significant variations, even after analysis of selected CpG sites from infection-associated genes (not shown), which indicated that global methylation of DNA does not change during influenza virus infection in this system.

#### Expected histone modifications in influenza virus infected cells

Histone fractions were purified from the same samples used for the study of DNA methylation and mass spectrometry (MS) analysis were performed. We used unbiased analysis with the nanoLC-MS platform to identify and quantify influenza virus-induced epigenetic changes in histone post-translational modifications (PTM). Three biological replicates were analyzed and quantitative analyses completed (see Methods). We considered a Mascot score threshold of 25 as high-confidence peptide identifications, and all peptides methylated/acetylated on lysine (above or below this threshold) were validated manually to assure correct tandem mass spectra matches. Only PTMs that were present in at least two replicas are presented (Fig. 1C) and it has to be pointed out that all modified and unmodified peptides were identified reliably more than once. Results in infected cells showed a reduction in lysine acetylation of histone 3 and 4. This decrease would impair host cell expression, in accordance with the role of acetylation in opening the condensed chromatin and activating transcription^17^. Non-methylated H3K36 and non-acetylated H4K79 levels increased moderately during infection; since H3K36 methylation and H4K79 acetylation are hallmarks of transcription elongation^18,19^, the increased levels of these unmodified residues would also contribute to transcriptional inactivation of host cells.

Histones have a large proportion of basic residues. This property impedes detection of specific residues, probably due to the presence of another lysine(s) that trypsin proteolysis would render as peptides too small to be identified reliably by MS-based techniques. Lysines not detected by MS include H3K4 and H4K20, whose PTM are recognized as histone marks that control cell transcription. H3K4 trimethylation is a common modification in active chromatin^20^, whereas H4K20 methylation is linked to transcriptional repression^20,21^. Decreased H3K4me3 levels have been observed during influenza virus infection^16^; here we evaluated possible changes in methylated H4K20 and in acetylated H4K16, as the latter is a conventional histone marker of active chromatin^22^. Total extracts of A549 mock-infected or PR8hv-infected cells at high m.o.i. were collected at various times post-infection and levels of acetylated H4K16 and dimethylated H4K20 were tested by Western blot. The results indicated that at late times after infection, influenza virus triggers a decrease in H4K16ac and an increase in H4K20me2 (Fig. 1D), which could contribute to a general decrease in host cell transcription activity.

#### Unexpected histone modifications in influenza virus infected cells

In contrast to this general effect on histone modifications, which seems to impair transcription, we detected a clear increase in methylation of lysine 79 of histone 3, especially monomethylation (Fig. 1C). This result was unanticipated, since studies in *Saccharomyces cerevisiae* and in humans, H3K79 methylation levels correlated clearly with transcriptional activity^23^. As methylation of this lysine gave the highest score in MS analysis, we evaluated this increase by two additional methods. We purified histones from cells mock- or PR8hv-infected at high m.o.i.; at 8 hpi, H3K79me2 levels were evaluated by Western blot analysis (Fig. 1E) and a quantitative colorimetric method based on anti-H3K79me2 antibody detection (Epigentek) (Fig. 1F). Both techniques verified the H3K79 methylation increase observed in proteomic analysis.

These results indicate that epigenetic modifications induced by influenza virus infection mainly target the histone component of host cell chromatin, with H3K79 residue methylation the most frequently modified. That methylation of infected cell DNA is unaffected indicates, that in these conditions, influenza virus does not permanently injure the host cell, but the epigenetic modifications may be transitory.

### Inhibition of H3K79 methylation increases influenza virus replication

Although most histone methyltransferases modify different lysines, only one known methyltransferase, Dot1L, mono-, di- and trimethylates H3K79^24^; the inhibitor EPZ-5676 (EPZ) specifically inhibits Dot1L. To confirm this ability to inhibit H3K79 methylation in our system, we incubated A549 cells with the inhibitor and used Western blot to analyze H3K79me2 accumulation at various times after EPZ addition. Treatment with 1 or 2 μM EPZ substantially decreased H3K79 methylation at 24–48 h post-EPZ addition, without affecting H3K4me3 levels (Fig. S1A). The effect of Dot1L inhibition on cell viability was analyzed by MTT assay (see Methods and^25^); at these doses, EPZ did not notably affect cell viability, even at 72 h after treatment (Fig. S1B).

**Figure 2:**
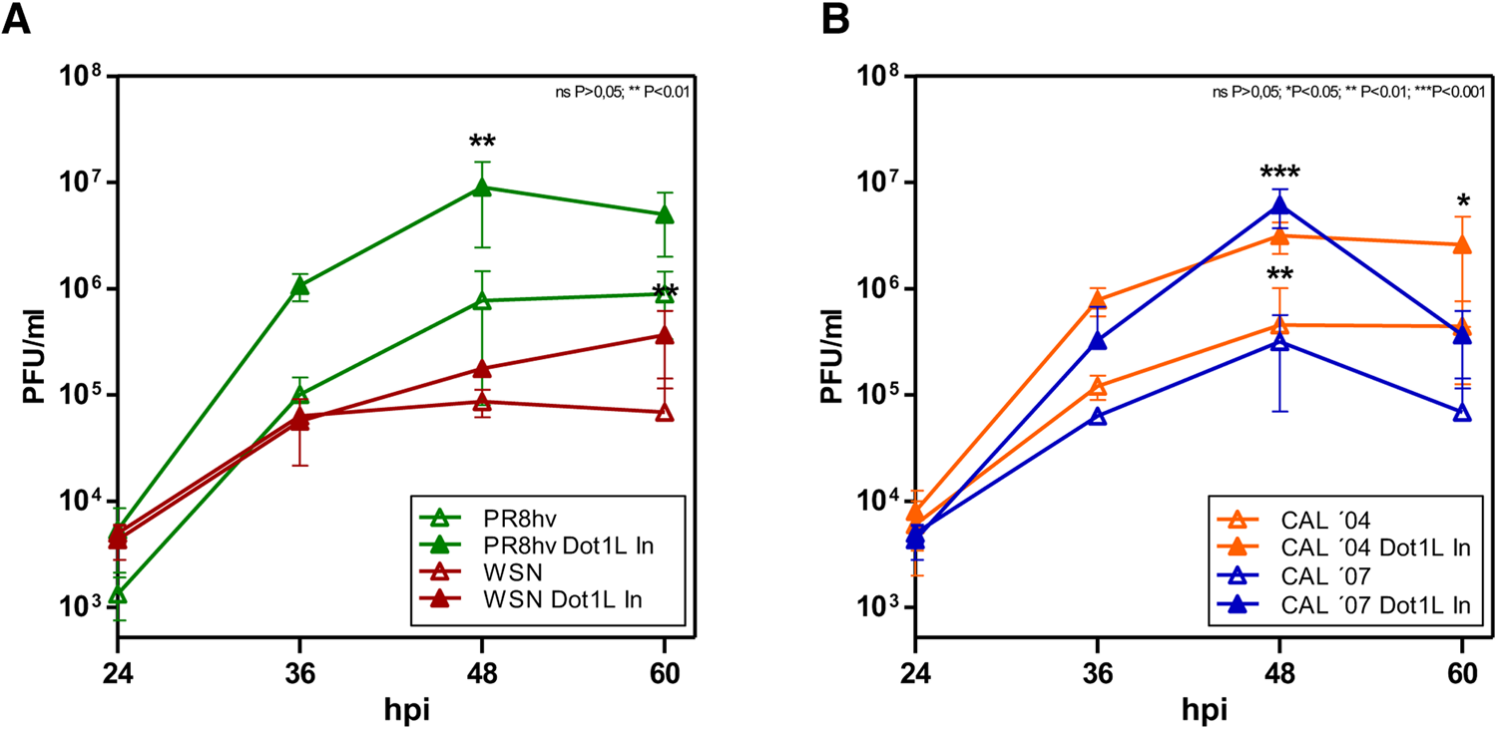
Influenza virus replication increases after specific inhibition of H3K79 methylation. A549 cells were plated alone or with Dot1L inhibitor (Dot1L In; 1 μM); after 48 h, they were infected with different strains at m.o.i. 10^−3^, the inhibitor was present throughout the infection in treated cells. Virus titer was determined by plaque assay on MDCK cells at indicated hpi. A549 cells were infected with PR8hv and WSN (**A**), or with CAL 04 and CAL 07 (**B**). Virus titer was determined by plaque assay on MDCK cells at indicated times post-infection. Student’s *t* test with Welch’s correction; 3 technical replicates of 3 independent experiments were performed.

To determine whether H3K79 methylation alters influenza virus infection, we inhibited Dot1L and analyzed its effect on production of infectious particles. A549 cells were plated alone or with Dot1L inhibitor (48 h), then infected at low m.o.i. with the PR8hv strain, with another laboratory-passaged influenza strain, A/WSN/33 (WSN) (Fig. 2A) or with one of two natural 2009 pandemic isolates, A/California/04/2009 (CAL04) and A/California/07/2009 (CAL07) (Fig. 2B) and analyzed production of infectious particles in each condition. Dot1L inhibition caused an increase in viral replication, higher in cells infected with the natural isolates, which suggests a general role of H3K79 methylation in control of the influenza virus life cycle.

### Downregulation of Dot1L increases influenza virus replication

**Figure 3:**
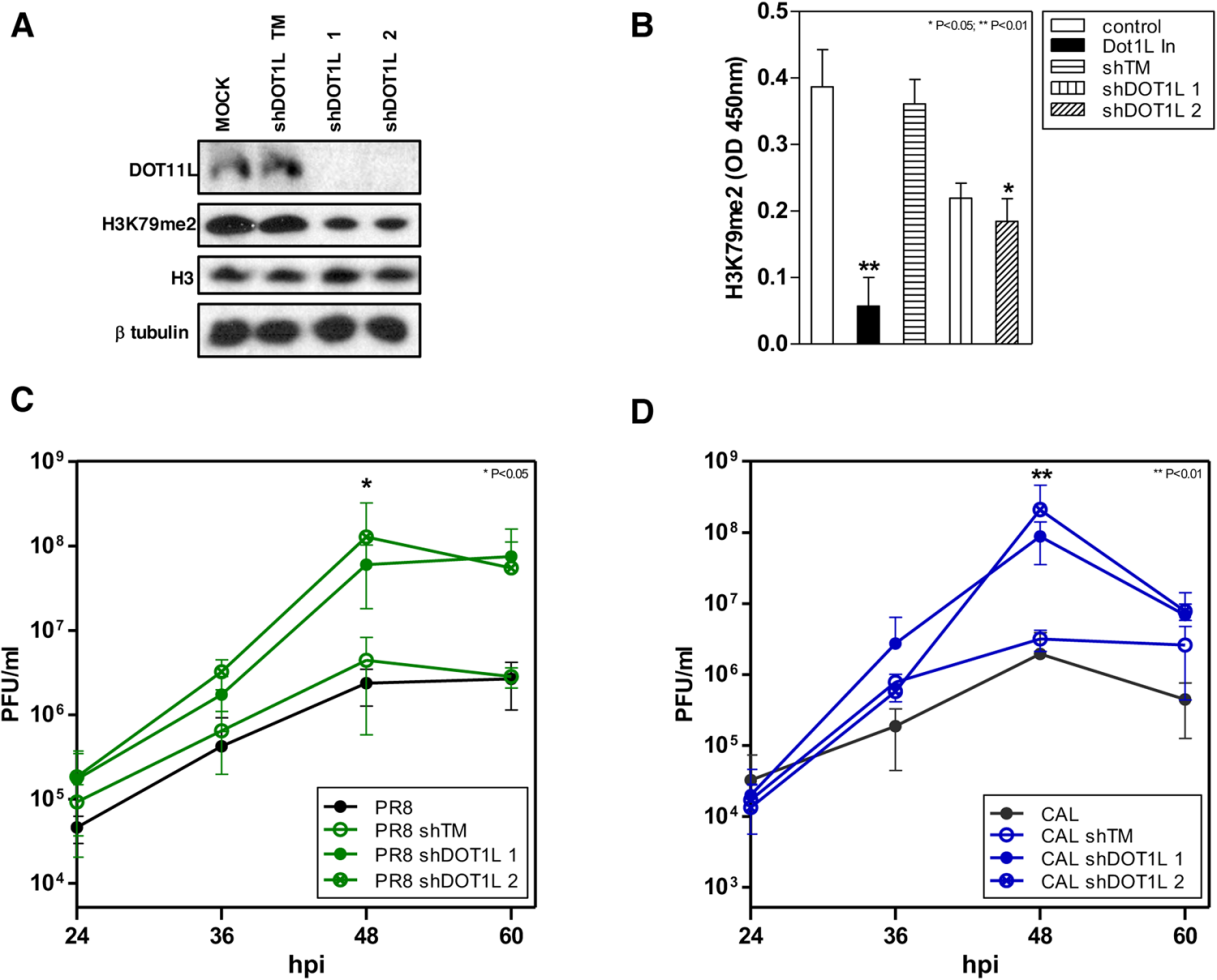
Influenza virus replication increases in Dot1L downregulated cells. A549 cells were treated with Dot1L inhibitor (48 h) or transduced with lentiviruses expressing shRNA specific for Dot1L (shDot1L 1, shDot1L 2) or an irrelevant shRNA (shTM) (5 days). The H3K79 methylation levels were analyzed by Western blot (**A**), or colorimetric analysis (**B**). A549 cells, uninfected or transduced with control or specific Dot1L silencers during 5 days, were infected with PR8hv (**C**) or CAL07 (**D**) strains at m.o.i. 10^−3^. Virus titer was determined by plaque assay on MDCK cells at indicated hpi. Student’s *t* test with Welch’s correction; 3 technical replicates of 3 independent experiments were performed.

To confirm the effect of Dot1L activity on influenza virus replication, we tested the effect of Dot1L knock-down on production of infective particles. For RNAi-mediated Dot1L silencing experiments, we used lentiviruses expressing shRNA specific for Dot1L (shDot1L 1, shDot1L 2) or a control that expresses irrelevant shRNA (shTM)^7,26^. Treatment of A549 cells with the Dot1L inhibitor or expression of the Dot1L-shRNAs reduced H3K79me2 levels compared with shTM, measured by Western blot analysis (Fig. 3A) and quantitative colorimetric method (Fig. 3B). Dot1L silencers decreased H3K79 methylation in infected cells in a lesser extent than EPZ treatment.

To analyze the relevance of Dot1L in virus replication, we transduced A549 cells with lentiviruses expressing the Dot1L silencers or control shRNA (5 days), followed by infection with the PR8hv (Fig. 3C) or CAL07 (Fig. 3D) strains at low m.o.i. In agreement with the results observed with the Dot1L inhibitor, Dot1L downregulation produced an increase in viral replication. These results reinforce the role of H3K79 methylation controlling influenza virus replication.

### Role of H3K79 methylation in antiviral responses

The above results suggested that modification of lysine 79 of histone 3 may mediate the antiviral response; its demethylation could decrease the host response against the pathogen allowing higher production of infectious particles. In agreement with this proposal we did not find significant reduction of viral titer in cells infected at high m.o.i where IFN signaling does not play a major role, in the presence of the Dot1L inhibitor (data not shown). These data suggested that H3K79 methylation would affect host response in conditions close to natural infections that take place in the epithelium of the respiratory tract at low multiplicity of infection and trigger an efficient IFN induction.

NF-κB is a protein complex that controls DNA transcription, cytokine production and cell survival. NF-κB is involved in cell responses to stimuli such as viral antigens and has a key role in regulating the innate immune response to infection^27^. NF-κB has been shown to be activated upon accumulation of influenza virus RNA^28^. In response to specific stimuli, NF-κB translocates from cytoplasm to the nucleus, where it binds promoters of regulated genes, activates transcription of the interferon pathway^29^, and stimulates ISG transcription. Type I interferon, which includes the IFNα and β subtypes, is induced during influenza virus infection and has an essential role in host defense against the virus by activating expression of a large number of ISG^30^.

**Figure 4:**
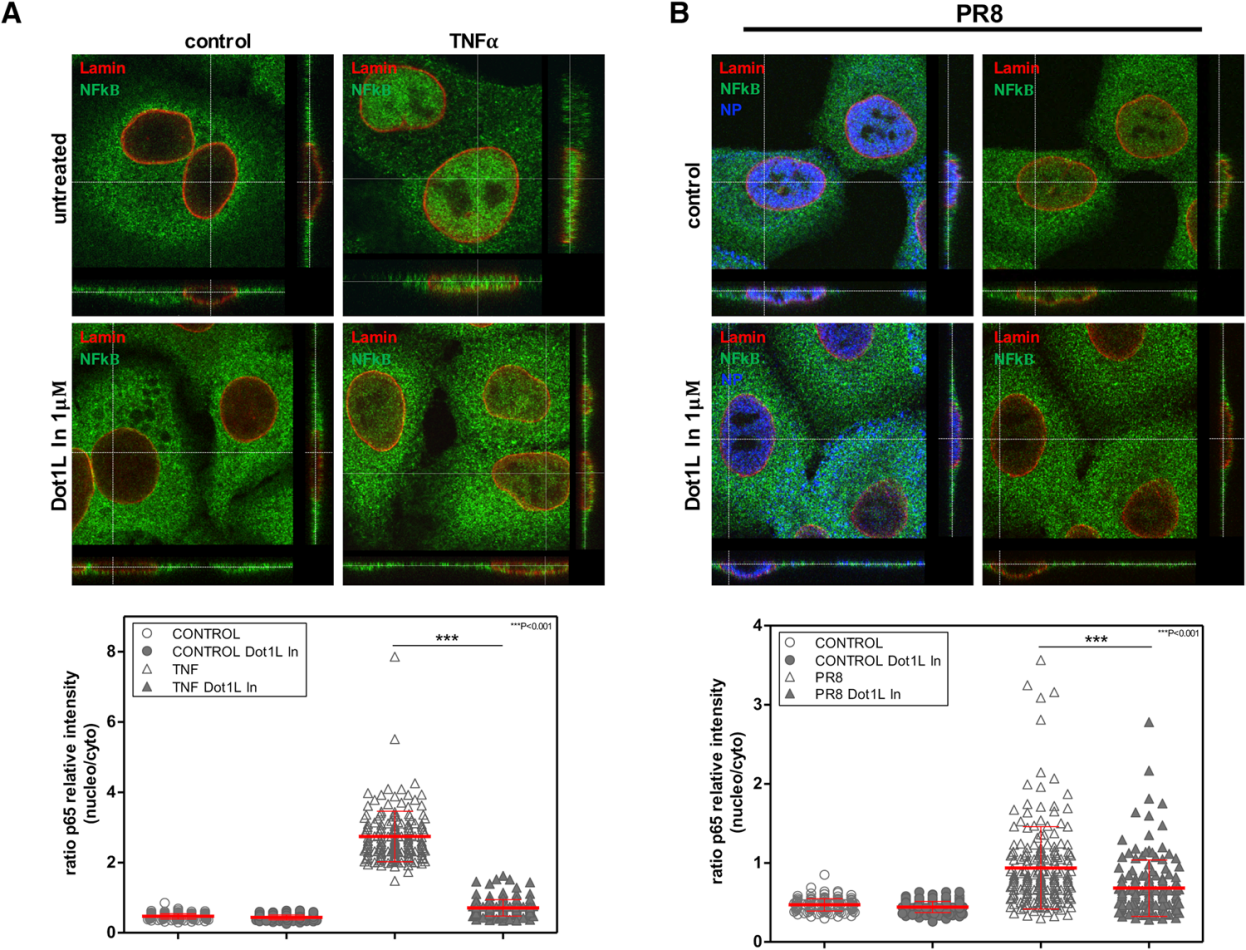
Treatment with Dot1L inhibitor decreases nuclear translocation of NF-κB. (**A**) A549 cells were plated alone or with Dot1L inhibitor (1 μM); 48 h later, TNFα was added (10 ng/ml, 30 min). Cells were processed for immunofluorescence using anti-lamin and -p65 antibodies. (**B**) Cells were PR8hv-infected (m.o.i. 3, 8 h) with or without Dot1L inhibitor as in part (**A**). The inhibitor was present throughout the experiment in treated cells. The cells were processed for immunofluorescence using anti-NP, -lamin and -p65 antibodies. Nuclear NF-κB translocation was measured by orthogonal projection image analysis and quantified in at least 200 cells/condition. The ratio was calculated of p65 relative intensity in the nucleus and cytoplasm of each cell in different conditions. Ratios are shown in dispersion graphs beneath representative images for each experiment. Three independent experiments were performed.

To analyze the effect of Dot1L methylase in NF-κB activation and IFN signaling, we evaluated NF-κB subcellular distribution in cells treated with tumor necrosis factor (TNF)-α (10 ng/ml, 30 min), or infected with influenza virus (1 PFU/ml, 8 hpi), alone or with Dot1L inhibitor (48 h). After TNF-α binding to the receptor, the inhibitory protein IκBα, which normally binds NF-κB and inhibits its translocation, is phosphorylated; this is followed by ubiquitination and degradation, releasing NF-κB, which is then translocated to the nucleus^29^. NF-κB distribution was monitored using antibodies to the p65 subunit of the NF-κB complex^31^ and anti-lamin A/C antibodies to identify the nuclear envelope; influenza NP detection was used to monitor infection. We analyzed >200 cells to quantitate nuclear and cytosolic NF-κB distribution (Fig. 4). TNF-α addition produced a significant increase in nuclear import of NF-κB, and Dot1L inhibitor treatment significantly decreased its nuclear translocation (Fig. 4A). Influenza virus infection similarly induced NF-κB nuclear import, and inhibition of H3K79 methylation reduced its nuclear translocation (Fig. 4B).

**Figure 5:**
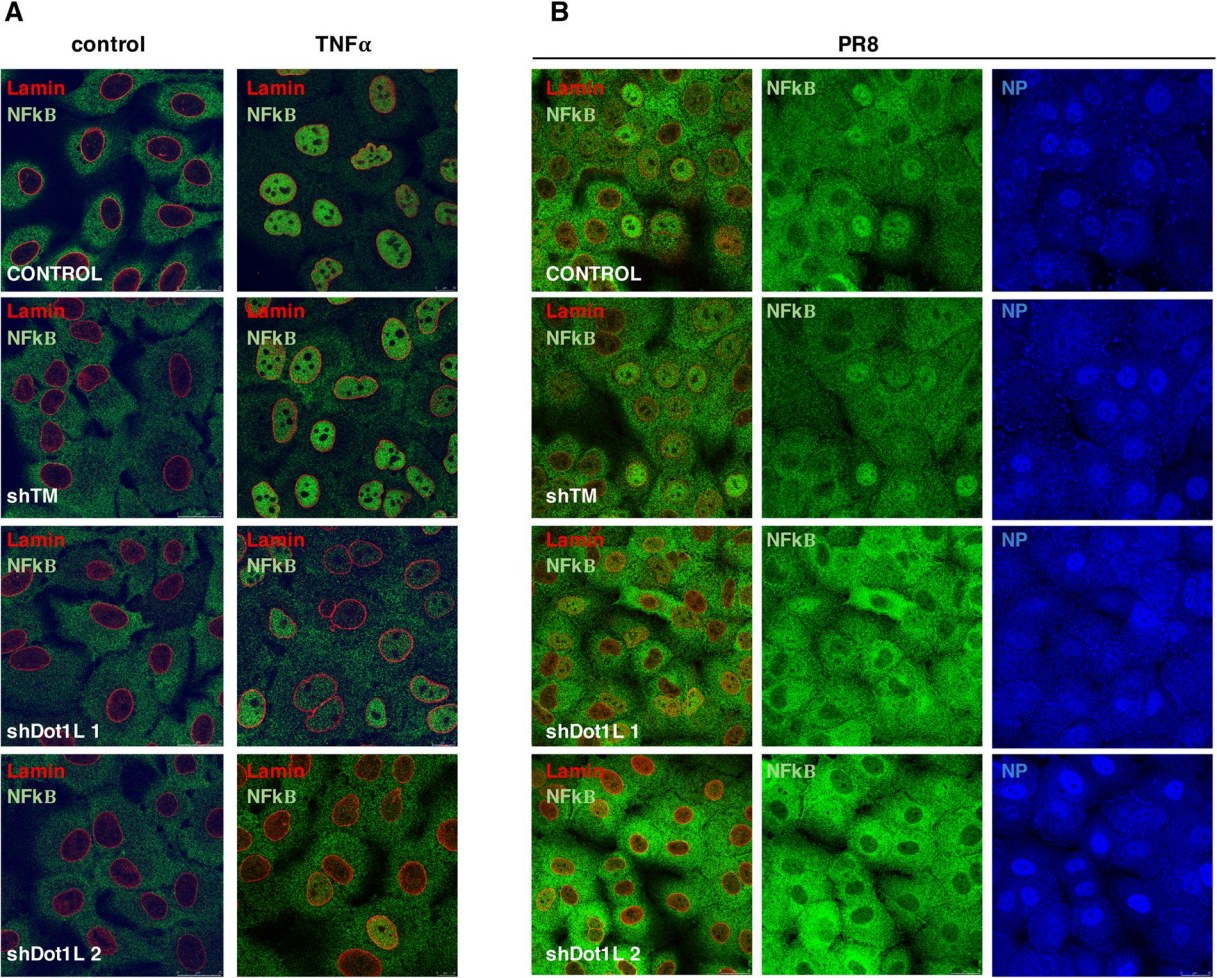
Downregulation of Dot1L decreases nuclear translocation of NF-κB. A549 cells were used as control (control), or transduced with lentivirus expressing an irrelevant shRNA (TM) or specific Dot1L silencers (shDot1L 1, shDot1L 2). 5 days later, TNFα was added (10 ng/ml, 30 min). Cells were processed for immunofluorescence using anti-lamin A/C and -p65 antibodies. (**B**) Cells were processed as in (**A**) and PR8hv-infected (m.o.i. 3, 8 h). Immunofluorescence analysis were carried out using anti-NP, -lamin and -p65 antibodies. Three independent experiments were performed.

Subcellular distribution of NF-κB was additionally examined in cells previously transfected with lentiviruses that express the specific Dot1L silencers or the control (5 days). Using the conditions described above, the corresponding cells were treated with TNF-α, or infected with PR8hv influenza virus. In agreement with the results obtained using the Dot1L inhibitor, the Dot1L silencers decreased the nuclear translocation of NF-κB both, in TNF-α treated cells (Fig. 5A) or in influenza virus infected cells (Fig. 5B), whereas the expression of the control silencer did not change the subcellular distribution of NF-κB. Together these results indicate that methylation of H3K79 modulates the pathway involved in the nuclear translocation of NK-κB.

**Figure 6:**
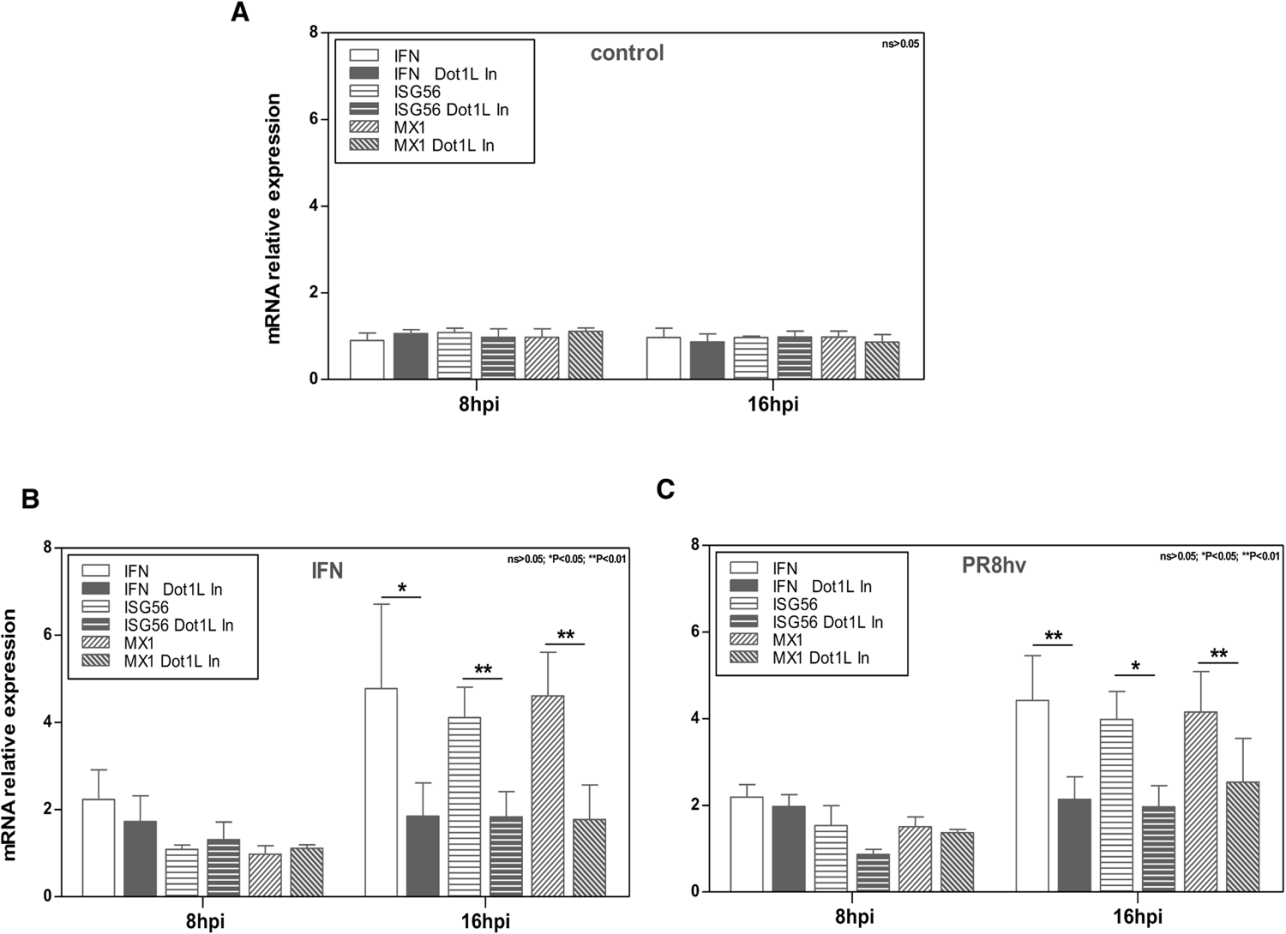
H3K79 methylation controls antiviral response. A549 cells were plated alone or with Dot1L inhibitor (Dot1L In, 1 μM, 48 h), then left untreated (**A**), treated with 100 U/ml IFNαβ (**B**) or PR8hv-infected (1 PFU/cell) (**C**) during 8 or 16 h. RNA was extracted and used for qPCR detection of IFNβ, ISG56 and MX1 genes. IFNαβ treatment was used as positive control and induced a significant increase in the genes analyzed (***P < 0.001). Student’s *t* test with Welch’s correction; 3 technical replicates of 3 independent experiments were performed.

As Dot1L inhibition impaired NF-κB activation, we examined its effect on steps that follow its nuclear translocation, that is, IFN and ISG RNA production. A549 cells were left untreated (control), treated with 100 U/ml IFNαβ (IFN) or were infected with PR8hv strain (1 PFU/cell) (PR8hv), alone or with Dot1L inhibitor (48 h). After 8 h or 16 h of IFN treatment or virus infection, RNA was extracted and used for qPCR detection. At 8 h, we found a weak increase on IFNβ, IFN-stimulated gene 56 (ISG56) and interferon-induced protein Mx1 (Mx1) RNA levels after IFNαβ addition or influenza virus infection, and Dot1L inhibitor treatment did not significantly decreased their accumulation (Fig. 6B,C). At 16 h, we observed an increase in IFNβ, ISG56 and Mx1 RNA levels and a reduction of these RNAs when cells were pretreated with the H3K79 methylase inhibitor (Fig. 6B,C). The significant reduction in NF-κB nuclear translocation and decreased IFNβ, ISG56 and Mx1 RNA expression after inhibition of Dot1L both, support a role for H3K79 methylation in modulating the antiviral response.

### The antiviral function of H3K79 methylase is not observed in IFN pathway deficient cells

**Figure 7:**
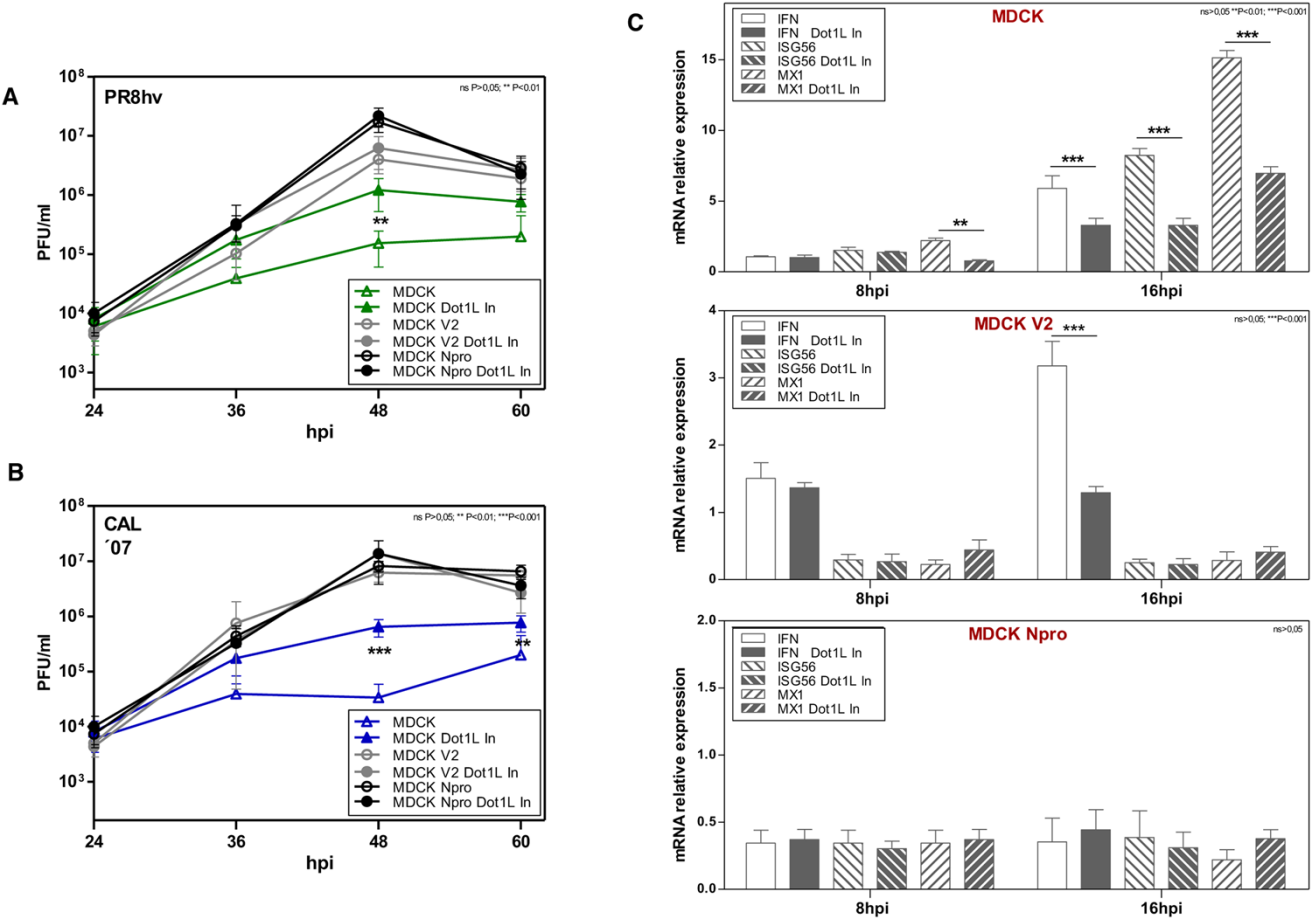
H3K79 methylation is involved in the IFN response to influenza virus. MDCK, MDCK V2 or MDCK Npro cells were plated alone or with Dot1L inhibitor (1 μM, 48 h), then infected (m.o.i. 10^−3^) with PR8hv (**A**) or CAL 07 (**B**). Virus titer was determined by plaque assay on MDCK cells at various hpi. In all cases, inhibitor was present throughout the experiment in treated cells. (**C**) MDCK, MDCK V2 or MDCK Npro cells were plated alone or with Dot1L inhibitor (1 μM, 48 h), then PR8hv-infected (1 PFU/cell). At indicated hpi, RNA was extracted and used for qPCR detection of IFNβ, ISG56 and MX1 genes. Student’s *t* test with Welch’s correction; 3 technical replicates of 3 independent experiments were performed.

Given the role of H3K79 methylation in the control of IFN signaling, we analyzed the effect of Dot1L inhibitor on influenza virus replication in cells with normal or deficient IFN responses. We used PR8hv and CAL07 strains at low m.o.i. to infect MDCK, MDCK V2 or MDCK Npro cells, untreated or treated with Dot1L inhibitor. MDCK V2 cells express V2 protein, which targets STAT1 and 2 for degradation^32^, and MDCK Npro cells express Npro protein, which targets IRF-3 for polyubiquitination and subsequent degradation^33^; IFN, but not ISG, is thus induced in MDCK V2 cells, and neither IFN nor ISG are induced in MDCK Npro cells. We analyzed viral titers at different hpi and observed that the increased infectious particle production in MDCK cells treated with the inhibitor and infected with PR8hv or CAL07 (Fig. 7A and B) was reduced or lost in ‘IFN-compromised’ cells treated with Dot1L inhibitor. The effect of influenza virus infection on H3K79 methylation in MDCK cells with normal and deficient response to IFN was checked by colorimetric assays. Influenza virus infection increased H3K79 methylation levels in normal and interferon deficient cells, likewise in A549 cells (Fig. S2A).

Since H3K79 methylation does not affect influenza virus replication in cells with impaired IFN signaling, we analyzed the effect of Dot1L inhibitor in subsequent stages of viral infection. We evaluated IFNβ, ISG56 and Mx1 RNA amounts after influenza virus infection (1 PFU/cell) in IFN-competent or -deficient cells, untreated or treated with Dot1L inhibitor. Influenza virus infection increased IFNβ, ISG56 and Mx1 production in MDCK control cells, and inhibitor treatment reduced their accumulation (Fig. 7C, top). Infection of MDCK V2 cells increased IFNβ production, which was reduced by Dot1L inhibitor, whereas accumulation of ISG was not observed, in accordance with the need for STAT signaling for their induction (Fig. 7C, center). Infection of MDCK Npro cells did not increase IFNβ, ISG56 or Mx1 RNAs, independently of the presence of Dot1L inhibitor (Fig. 7C, bottom), which coincided with the inability of these cells to produce IFN and ISG. In addition, total extracts of MDCK, MDCK V2 and MDCK Npro cells infected with PR8hv at 3 m.o.i for 8 h were used for Western blot analysis to detect IRF-3, STAT1, Mx and ISG56 to confirm the differential expression of these proteins in normal or IFN-deficient cells (Fig. S2B).

These results indicate that the interferon antiviral response to influenza virus infection is at least partially regulated by H3K79 methylation via canonical type I IFN signaling mediated by the STAT pathway at the level of IFN activation and response.

### H3K79 methylation modulates replication of interferon-inducers viruses

These results indicated the ability of Dot1L methylase to control interferon signaling pathways. To evaluate whether H3K79 methylase has a broader role in the control of virus multiplication, we tested the effect of Dot1L inhibitor and the lentiviruses expressing the Dot1L silencers on the replication of two distinct RNA viruses, respiratory syncytial virus (RSV) and vesicular stomatitis virus (VSV). Like influenza virus, RSV is a respiratory virus, and is the most common cause of viral lower respiratory tract infections in infants and children^34^. Although IFNβ appears to restrict viral replication in lung epithelial cells, it is generally agreed that RSV is relatively resistant to IFNαβ, effects, and IFN has not been detected in natural infection^34^. VSV can infect insects, cattle, horses and pigs and is a potent IFN inducer both in cell culture and animal models^35^.

**Figure 8:**
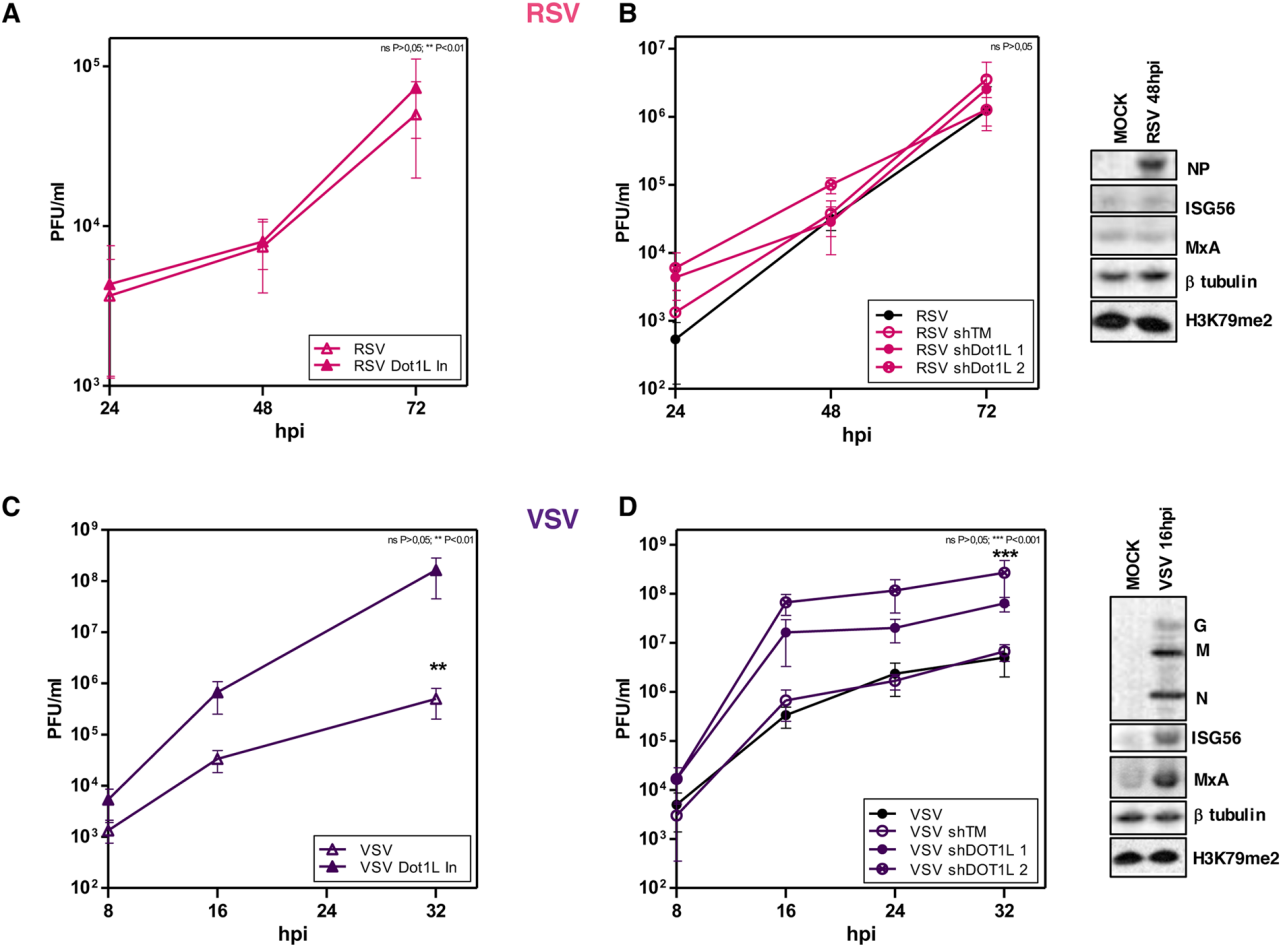
H3K79 methylation modulates replication of interferon-inducers viruses. A549 cells were plated alone or with Dot1L inhibitor (1 μM, 48 h) (**A**,**C**)), or transduced with lentiviruses expressing a control shRNA (TM), or specific Dot1L silencers (shTM, shDot1L 1, shDot1L 2, 5 days) (**B**,**D**). After that, the cells were infected with RSV (m.o.i. 1) (**A**,**B**) or VSV (m.o.i. 10^-3^) (**C**,**D**). The inhibitor was present throughout the experiment in treated cells. Right panels show the corresponding expression of viral proteins and the indicated proteins measured by Western blot analysis. Virus titer was determined by plaque assay at indicated times post-infection. Student’s *t* test with Welch’s correction; 3 technical replicates of 3 independent experiments were performed.

Human epithelial A549 cells, untreated or treated with Dot1L inhibitor (48 h) (Fig. 8A,C), or transduced with lentiviruses expressing the Dot1L silencers or control shRNA (5 days) (Fig. 8B,D), were infected at low m.o.i. with RSV (1PFU/ml) (Fig. 8A,B) or VSV (10^−3^ PFU/ml) (Fig. 8C,D); at various times post-infection, cell supernatants were obtained for viral titration (see Methods). No changes were observed in RSV replication in the presence of the inhibitor or the Dot1L silencers, whereas infectious particle production clearly increased in VSV-infected cells treated with Dot1L inhibitor or when Dot1L was down-regulated through the expression of the specific silencers. We also evaluated the expression of viral and ISG proteins (ISG56 and MxA) during infection with RSV (Fig. 8B, right), and VSV (Fig. 8D, right). The corresponding viral proteins were detected and we observed a lack of IFN-stimulated gene induction in RSV-infected cells, while infection with VSV efficiently induced IFN response in our system. These results support a role for H3K79 methylation in the control of IFN signaling, and a potential general Dot1L methylase function in regulating pathogen infection controlled by the IFN pathway.

## Discussion

Viruses are obligate intracellular parasites that completely subordinate host cell metabolism. Although influenza virus does not integrate into the genome of the infected cell, the virus efficiently switches off expression of host cell genes^36^, a result of a complex interplay of virus-induced activities closely coordinated to reduce the host response to eliminate the viral infection. Consequently, during infection there is a network of viral- and host-induced modifications of cellular gene expression with a close dependence of chromatin-based functions and therefore of chromatin dynamic. Accordingly, specific interactions between chromatin remodelers of the CHD family and influenza virus proteins have been described such as the association of CHD3 with the non-structural protein NS2^37^ or CHD6 and CHD1 with the viral polymerase complex^16,38,39^. In spite of this association, little effort has been made to identify epigenetic changes in chromatin induced by the infection.

Few alterations in DNA methylation have been reported during influenza virus infection^9–11^. Among them changes in promoter methylation levels of some proinflammatory cytokines and interleukine genes when using the high virulence H5N1 strain have been described. Infection of human respiratory epithelial cells with PR8hv strain did not show variations on DNA methylation (Fig. 1B), however it is possible that changes in promoter methylation of specific genes and their subsequent inactivation, take place in response to particular virulent influenza strains and/or in different systems.

Previous studies showed that influenza virus infection modulates PTM of several ISG. Chromatin immunoprecipitation assays and antibodies that recognize canonical marks of transcription activation such as H3K4me3 or of inactivation such as H3K27me3 showed addition or removal of these PTM in several ISG; modifications depended on strain virulence when H5N1 virus was compared with the H1N1 2009 pandemic strain^8^. This study emphasized the importance of epigenetic control in the antiviral response elicited by influenza virus infection; nonetheless, they focused on the search for specific canonical transcription activation or repression marks of histones that correlate with up- or down-transcriptional regulation of the corresponding genes and do not provide an overview of all possible changes to chromatin in response to the viral infection.

For a broad overview of chromatin modifications in influenza virus-infected cells, we used an unbiased search for global changes in chromatin epigenetics at the DNA and histone levels. We observed a general decrease on histone acetylation in H3 and H4 lysine residues after infection, as well as increased levels of unmodified H3K36, H4K79 and dimethylated H4K20 (Fig. 1C and D). In addition, previous studies showed reduction of H3K4me3; one of the hallmark of active chromatin^16^. Histone acetylation has a key role opening the condensed chromatin, resulting in charge neutralization and a more relaxed, open, and transcriptionally active chromatin structure^17^. Decreased acetylated histones, trimethylated H3K4 and increased non-methylated H3K36 and dimethylated H3K20, all associate with transcriptional inactivation^18–21^. These epigenetic changes would impair host cell expression, in accordance with the transcription inactivation of the host cell that occurs during infection^40^ and constitutes one of the major mechanisms triggered by the virus to inactivate host transcription machinery and consequently to decrease the antiviral response.

Unexpectedly, changes in methylation of lysine 79 of histone 3 were the most prominent. Lysine 79 is located within the globular domain of histone H3 and is mono-, di-, and trimethylated by Dot1L, which modifies this lysine exclusively^24^. H3K79 methylation is increased in actively transcribing genes and appears to have a role in cell cycle regulation and DNA damage response^41^. Dot1L has been studied particularly in the modulation of mixed-lineage leukemia (MLL)-related leukemogenesis, as MLL fusion target gene expression depends specifically on the functional DOT1L gene^42^; indeed, inhibition of Dot1L activity is currently in clinical trials.

The role of H3K79 methylation in viral infection, is poorly characterized. Infection of human primary fibroblasts with human cytomegalovirus (HCMV), produces a marked increase in H3K79me2 levels and downregulation of Dot1L expression results in a decreased viral growth^43^. The genome of HCMV is a double-stranded DNA molecule that is maintained as episome during infection. The viral DNA lacks histones when encapsidated in the virion however, upon infection the viral genome is transported to the nucleus, where it becomes associated with host cell histones. It has been speculated that Dot1L directly mediates replication of the HCV genome, in agreement with the chromatinization of its DNA genome^44^.

The function of H3K79 methylation modulating influenza virus replication, suggest a very different mechanism, since influenza virus is an RNA virus that does not integrate in the host chromatin and its genome lacks histones. In physiological conditions where antiviral signaling is induced, decreased H3K79 methylation clearly enhanced viral replication (Figs 2 and 3). Moreover, nuclear translocation of NF-κB (Figs 4 and 5) and accumulation of IFNβ and ISGs (ISG56 and Mx1) decreased in influenza virus-infected cells with Dot1L downregulated (Fig. 6).

Influenza virus and vesicular stomatitis virus induce a strong antiviral immune response characterized by robust production of antiviral type I interferons. During infection, double-stranded RNA molecules are produced, which are recognized by the RIG-I helicase^45^. After activation, RIG-I recruits various TNF receptor-associated factors, which trigger phosphorylation and activation of the Iκκ complex, leading to IκBα phosphorylation and degradation to allow NFκB nuclear translocation^46^. NF-κB and IFN regulatory factor 3 direct expression of type I IFN, which promote transcription of a variety of genes that further limit viral replication^45,47^. We observed a marked increase on replication of influenza virus and VSV, both potent inducers of IFN, in cells with low levels of methylated H3K79 (Figs 2, 3 and 8). This effect is lost when cells deficient on IFN signaling are infected with influenza virus (Fig. 7).

Together the data indicate that demethylation of H3K79 would decrease antiviral IFN signaling and therefore, viruses may develop different strategies attempting to control epigenetic modification of H3K79 that elicit the anti-pathogen response. Methylation of lysine 79 of histone 3 might have a general role in controlling the host response of pathogens that are interferon inducers.

## Methods

Human respiratory cells (A549), Madin-Darby canine kidney (MDCK), MDCK V2 and MDCK Npro cells (provided by R. E. Randall) were cultured in Dulbecco’s modified Eagle’s medium (DMEM) with 10% fetal calf serum. The mouse-adapted influenza viruses A/PR78/34 of high pathogenicity (hvPR8)^13^, A/WSN/33 (WSN), A/California/04/2009 (CAL 04) and A/California/07/2009 (CAL 07) were used. Human respiratory syncytial virus (RSV) was provided by J.A. Melero and vesicular stomatitis virus (VSV) by R. Alfonso.

EPZ5676 was purchased from Novagen, human tumor necrosis factor α from Sigma-Aldrich, and recombinant interferon αβ (PBL 11200-2 universal type I IFN) was provided by S. Guerra.

### Lentiviral particle production and cell transduction

Lentiviral particles were produced in HEK293T cells by cotransfection of plasmids psPAX2 and pMD2.G with each of the pLKO-based shRNA vectors, as described^48,49^. Supernatants were collected 40 to 48 h post-transfection, filtered through a 0.45 μm filter, and used to transduce the corresponding cells. As the lentiviral vectors confer puromycin resistance, the minimum amount of supernatant necessary to confer 100% resistance to puromycin (5 μg/ml) was used. Silencing was tested by Western blotting or colorimetric assays, normally at 5 days post-transduction.

### Virus infection

Cultured A549, MDCK, MDCK V2 or MDCK Npro cells were infected at 1–3 PFU/cell (high m.o.i.) or at 1 × 10^−3^ PFU/cell (low m.o.i.) for all viruses except RSV (1 PFU/cell). After 1–2 h, non-bound virus was rinsed off and, at different times (hours post-infection; hpi), cell extracts were collected and used for virus titration by plaque assay or for Western blot. Cell culture infections were performed in BSL2 conditions.

### Western blot

Cells infected at indicated m.o.i. were collected at different hpi in Laemmli sample buffer. Western blot was performed as described^40^. To detect influenza virus proteins, the following antibodies were used: for PA, monoclonal antibodies (mAb) 2 and 9 (1:250;^50^; for PB2, mAb 22 (1:100;^50^, and for PB1, rabbit polyclonal antibody (1:1000;^51^; for β-tubulin, mouse anti-β-tubulin mAb (1:1000; Sigma). To analyze histones, we used H4K16ac ab109463 (1:1000; Abcam) and H4K20me2 (1:1000; Active Motif). Polyclonal antibodies to H3K79me2 D15E8, H3K4me3 C42D8, H4 #2592 and H3 D1H2 (1:1000) were from Cell Signaling. For VSV detection, a monoclonal antibody mix against G, N and M viral proteins provided by M. Esteban was used. For RSV detection, a monoclonal antibody against NP protein provided by J.A. Melero was used.

### Colorimetric determination of H3K79me2

The iQuik Global Di-Methyl Histone H3K79 Quantification Kit (Colorimetric) from Epigentek was used for ELISA-like measurement of total H3K79me2 amounts, following the supplier’s protocol.

### Proteomic analysis of histone modifications

#### Enzymatic digestion and iTRAQ-4plex labeling

The enriched histone fraction (30 μg) for each condition, prepared following the Epigentek protocol, was precipitated by the methanol/chloroform method, reconstituted in 7 M urea/2 M thiourea/100 mM TEAB buffer (triethylammonium bicarbonate, pH 7.5), reduced with 50 mM Tris(2-carboxyethyl) phosphine (TCEP, SciEx), and alkylated with 200 mM methyl methanethiosulfonate (MMTS, Pierce); this was followed by trypsin (Sigma-Aldrich) proteolysis at a 1:20 enzyme:protein ratio. The tryptic peptides were labeled using the iTRAQ-4plex Isobaric Mass Tagging Kit (SciEx) according to manufacturer’s instructions (mock, tag-114; PR8lv, tag-115, PR8hv, tag-116). Samples were pooled, dried and desalted on a SEP-PAK C18 cartridge (Waters).

#### LC-MS/MS triple TOF analysis

Peptide fractions were subjected to LC-MS/MS analysis using a nano liquid chromatography system (Eksigent Technologies nanoLC Ultra 1D plus) coupled to a high speed Triple TOF 5600 mass spectrometer (SciEx) with a nanoelectrospray ion source. Samples were injected on a C18 PepMap trap column (5 μm, 100 μm I.D. x 2 cm; Thermo Scientific) at 2 μL/min, in 0.1% formic acid in water, and the trap column was switched on-line to a C18 nanoAcquity BEH analytical column (1.7 μm, 100 Å, 75 μm I.D. x 15 cm, Waters), equilibrated in mobile phase A (0.1% formic acid in water), and peptide was eluted in a 120 min linear gradient from 5–40% B (0.1% formic acid in acetonitrile) at 250 nL/min. The mass spectrometer was operated in data-dependent acquisition mode. For TOF scans, accumulation time was set to 250 ms, and up to 15 precursor ions were monitored per cycle.

#### Data analysis

MS and MS/MS data obtained were converted to mgf files, which were also searched against a *Homo sapiens* protein database containing 138362 protein-coding genes (including reversed entries to calculate false discovery rate; FDR) using the Mascot Server v. 2.4 (Matrix Science). Search parameters were set as follows: enzyme, trypsin; allowed missed cleavages, 3; fixed modifications, beta-methylthiolation of cysteine and iTRAQ-4plex (N-term and K); variable modifications, oxidation of methionine, acetylation (K and N-term) and methylation and dimethylation (K). Peptide mass tolerance was set to ±25 ppm for precursors and 0.05 Da for fragment masses. The confidence interval for protein identification was set to ≥95% (p < 0.05) and only peptides with an individual ion score above the 1% FDR threshold were considered correctly identified.

### DNA methylation

Isolated DNA was processed according to the manufacturer’s protocols for Illumina Infinium Assay, as described^52^.

#### Data filtering

From the initial 485,577 CpG, we excluded all probes with detection p-values > 0.001 (1135 CpG removed). We eliminated CpG containing single-nucleotide polymorphisms (SNP) located within 10 bp of the target CpG (snp10) and internal controls (ch and rs), resulting in 39691 CpG removed. A total of 444,751 valid probes were included in the final analysis. Methylation score was represented as β-values, average for each probe was calculated and β-value differences were used for analysis.

### Confocal immunofluorescence microscopy

Cells were fixed in 4% paraformaldehyde (20 min, room temperature) and stored in PBS. For immunofluorescence, cells were permeabilized (5 min) in PBS containing 1% Triton X-100 and incubated with primary antibodies diluted in PBS/4% BSA (w/v) as follows: rat anti-NP (1:5000^53^;, anti-p65 ab16502 (1:500; Abcam) and anti-lamin A/C (636)(1:200; sc-7292, Santa Cruz) to label nuclear envelope. Confocal microscopy was performed with a Leica TCS SP5 laser scanning system. Images of 1024 × 1024 pixels and an eight-bit grayscale depth were acquired sequentially every 0.2–0.3 μm using LAS AF version 2.2.1 software (Leica) and analyzed using LAS AF and MetaMorph Premier version 7.5.2 image analysis software (Molecular Devices). p65 nuclear translocation was quantified by counting at least 200 cells/condition, and the ratio of relative p65 intensity in nucleus and cytoplasm of each cell was calculated.

### qRT-PCR analysis

For RNA extraction, cell pellets were resuspended in 1 ml TRIzol reagent (Invitrogen) and RNA was purified as recommended by the manufacturer. RNA was digested with RNAse-free DNAse (1 U/mg; 1 h, 37 °C), extracted with phenol-chloroform-isoamyl alcohol and ethanol-precipitated.

For reverse transcription, we used the High-Capacity cDNA RT kit (Applied Biosystems). PCR were performed in 96-well PCR plates using SYBR green PCR master mix (Applied Biosystems). PCR were carried out in a PRISM 7000 Sequence detection system (Applied Biosystems). The cycle threshold (Ct) was determined with analytical software (SDS; Applied Biosystems). Serial dilutions of cDNA were used to ensure amplification.

## Electronic supplementary material

Supplementary Information
