# Supplementary material for: Epigenetic control of influenza virus: role of H3K79 methylation in interferon-induced antiviral response
Source: Sci Rep. 2018 Jan 19;8:1230. doi: 10.1038/s41598-018-19370-6 (PMC5775356; doi:10.1038/s41598-018-19370-6)
Supplement: Supplementary file 1 — Supplementary Information [file 41598_2018_19370_MOESM1_ESM.pdf]

Epigenetic control of influenza virus: role of H3K79 methylation in interferon-induced antiviral response

Laura Marcos-Villar, Juan Díaz-Colunga, Juan Sandoval, Noelia Zamarreño, Sara Landeras-Bueno, Manel Esteller, Ana Falcón and Amelia Nieto

SFig.1

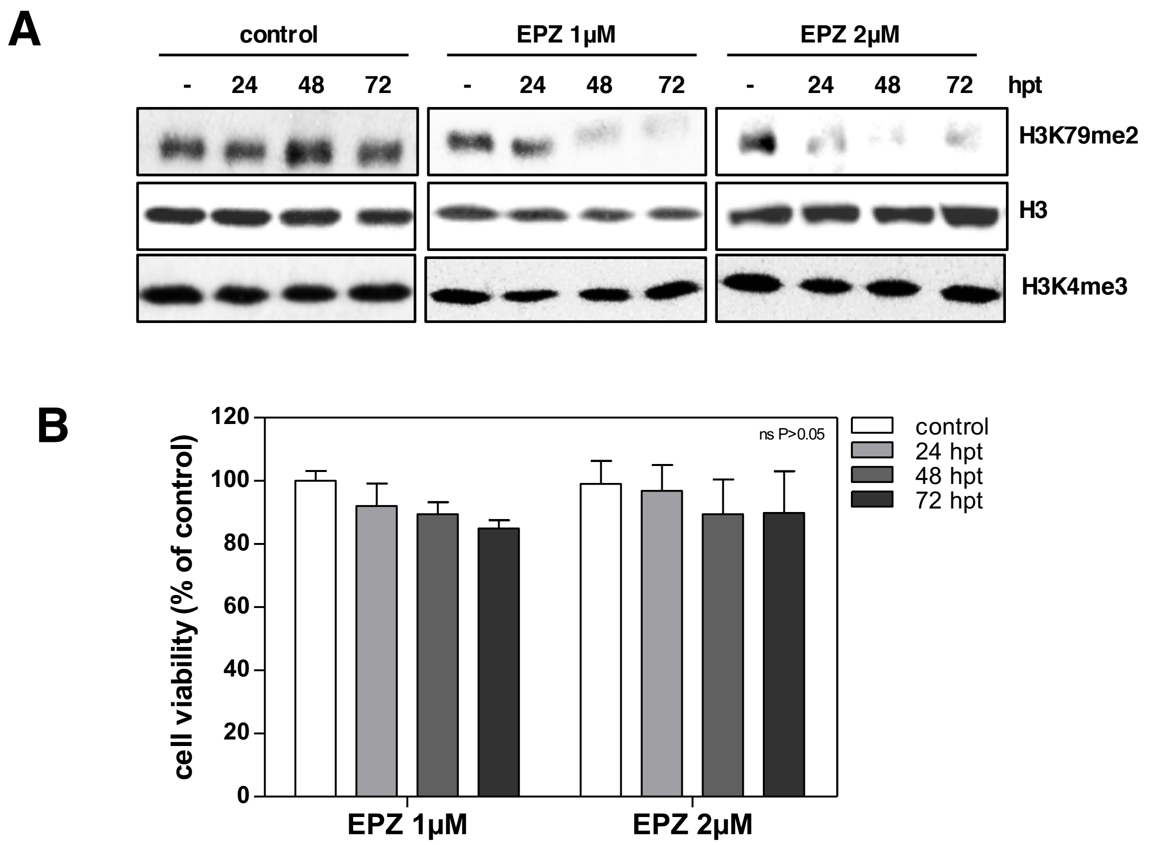

Supplemental Fig. 1. Specificity and cell viability of Dot1L inhibitor treatment. A) Total extracts of A549 cells treated with DMSO, 1 **μ**M or 2 **μ**M Dot1L inhibitor (EPZ) were collected at various times post-treatment; H3 and methylated H3K79 and H3K4 levels were determined by Western blot. B) Viability of A549 cells treated with Dot1L inhibitor was determined by MTT assay to measure cell metabolic activity.

Epigenetic control of influenza virus: role of H3K79 methylation in interferon-induced antiviral response

Laura Marcos-Villar, Juan Díaz-Colunga, Juan Sandoval, Noelia Zamarreño, Sara Landeras-Bueno, Manel Esteller, Ana Falcón and Amelia Nieto

SFig. 2

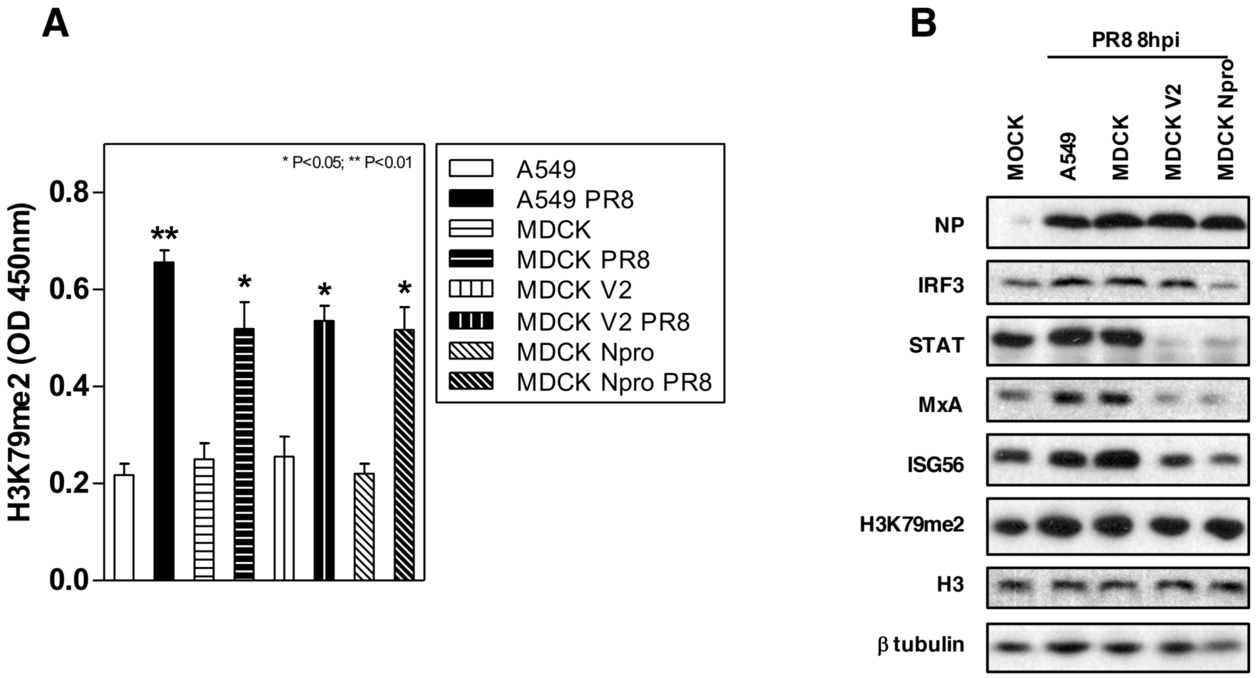

Supplemental Fig. 2. Accumulation levels of H3K79 and IFN-related protein in normal and cells deficient for IFN response. A) Cells were left uninfected or infected with influenza virus PR8 at 3 m.o.i for 8 h. H3K79 methylation levels, were analyzed by colorimetric assays (B). Uninfected A549 cells (MOCK), or A549, MDCK, MDCK V2 and MDCK Npro cells were infected with PR8hv (3 m.o.i., 8 h), total extracts were obtained and used for detection of the indicated proteins by Western blot assays.
